# 

**Published:** 1896-01-11

**Authors:** 


THE HOSPITAL. ABSOLUTELY PURE, therefore BEST. JANUARY 11TH, 1896.

CADBURY'S COCOA
The standard of highest purity at present attainable."—*Lancet.*

CADBURY'S COCOA

NO ALKALIES USED.

### THE HOSPITAL

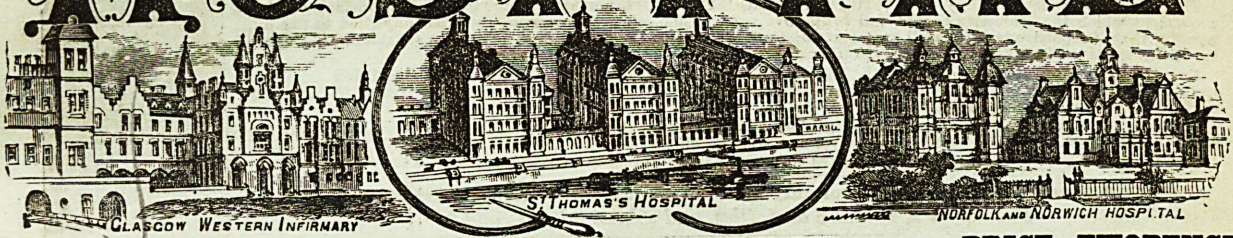

No. 485. Vol. XIX.

SATURDAY, JAN. 11TH, 1896.

WITH EXTRA NURSING SUPPLEMENT.

[Registered at the General Post Office as a Newspaper for transmission in the United Kingdom and Abroad.]

